# Analysing the genetic diversity of three sheep breeds in Turkey and nearby countries using 50 K SNPs data

**DOI:** 10.1080/10495398.2024.2329106

**Published:** 2024-03-18

**Authors:** Mervan Bayraktar

**Affiliations:** Department of Animal Science, Faculty of Agriculture, Çukurova University, Adana, Turkey

**Keywords:** Population structure, genetic distance, genetic differentiation, sheep breeds, inbreeding coefficient

## Abstract

This study analysed the genetic diversity and population structure of eight sheep breeds in Turkey and nearby countries. Moderate genetic diversity was observed, with the Sakiz (SKZ) exhibiting the highest diversity based on heterozygosity and allelic richness (AR) values. Genetic distances revealed differentiation between the populations, with the most significant divergence between the Cyprus Fat Tail (CFT) and SKZ breeds. PCA demonstrated SKZ and Chios (CHI) clustering together, indicating genetic similarity. Karakas (KRS), Norduz (NDZ), Afshari (AFS), Moghani (MOG) and others showed overlap, reflecting genetic relationships. Ancestry analysis found that KRS was predominantly inherited from the second ancestral population, while SKZ and NDZ were primarily derived from the first and second ancestral lineages. This illustrated the populations’ diverse origins. Most genetic variation (96.84%) was within, not between, populations. The phi-statistic (PhiPT) indicated moderate differentiation overall. Phylogenetic analysis further demonstrated the genetic distinctiveness of the SKZ breed. ROH and FROH analyses showed that SKZ exhibited the highest homozygosity and inbreeding, while KRS displayed the lowest. This study elucidates these breeds’ genetic diversity, structure and relationships. Key findings include moderate diversity, evidence of differentiation between breeds, diverse ancestral origins and distinct ROH patterns. This provides insights into the population’s genetic characteristics and conservation requirements.

## Introduction

Sheep are among the earliest species domesticated by humans, with archeological findings indicating their domestication occurred approximately 11,000 years ago in the Fertile Crescent, a region in the Near East. As a dependable resource, sheep have historically provided meat, milk, and wool and continue to be a vital livestock species worldwide.^1–10^ In Turkey, sheep husbandry boasts a lengthy tradition, and the country is recognized for its diverse native sheep breeds, each uniquely adapted to Turkey’s varied agroecological landscapes. Understanding these breeds’ genetic diversity and population structure is crucial for developing effective conservation strategies and genetic enhancement programs.^11^^,^^12^

**Figure 1.**
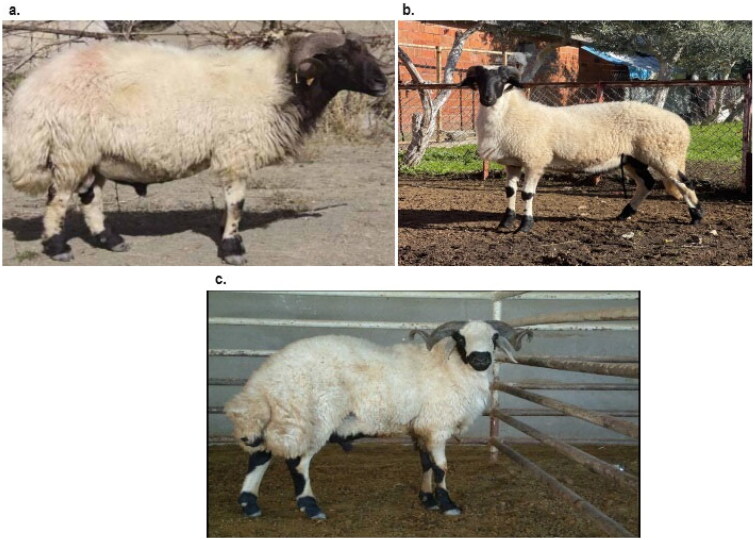
Comparative morphology of distinct sheep breeds: a) Norduz; b) Sakiz; c) Karakas.

One of the most common sheep in Turkey is the Akkaraman breed, which constitutes about (43–45%) of Turkish sheep. There are many types of this breed, including Karakas (KRS) and Norduz (NDZ) sheep. The KRS sheep, a variety of the Akkaramans, are predominantly bred in Van Province and neighboring provinces. They have a distinct color pattern – black and white heads with cream-colored bodies and black markings. KRS are agile and often graze alongside goats. They are bred mainly for lamb production, with some secondary milk yield for cheesemaking. The KRS are well-adapted to the harsh continental climate and local diseases. This adaptability makes it a preferred breed in the region (Fig. 1).^13–15^ The NDZ is a variety of the Akkaraman breed found in mountainous border regions of Turkey and Iran. They are large sheep with three-part fat tails and primarily white coats with some ash coloring and black markings. NDZ males commonly have horns, while fewer females are horned. They are kept in large flocks due to high milk production, fast growth rates, adaptability to cold climates and disease resistance. The average milk yield is 135–140 kg over a 180-dy lactation. The NDZ is an economically significant breed valued for its productivity and hardiness.^16^^,^^17^ The Chios breed originates from the island of Chios and is found along the Aegean coastal region of Turkey. Chios sheep have distinctive black and white facial and leg markings and long, fatless tails. They are kept in small household flocks of 3–5 animals. The breed is known for its high milk yield. However, Chios sheep adapt poorly when raised outside their native environment. The Chios has the highest milk yield among Turkish breeds, but its meat is less flavorful than the Akkaraman and Daglic breeds.^18^ Sheep production significantly impacts Turkey’s agricultural economy, supporting food security, income generation and employment in rural communities. The diversity of Turkish sheep breeds reflects the country’s diverse landscapes, climates and farming systems. These breeds are valued for their high fertility, disease resistance and the quality of their meat, milk and wool, especially in low-input pastoral systems.^19^,^20^ However, native Turkish sheep breeds face challenges, including genetic erosion due to changing agricultural practices, crossbreeding, and the introduction of exotic breeds. Comprehensive genetic analyses using molecular tools are essential for understanding population structure, inbreeding, and genetic erosion. This knowledge is crucial for setting conservation priorities, utilizing breed-specific traits, and implementing coordinated breeding programs that incorporate genetic and genomic information.^21–23^ Maintaining genetic diversity is essential for the long-term sustainability of the Turkish sheep industry. While crossbreeding with exotic breeds can increase productivity, it often leads to a decline in native breeds. Strategic breeding programs, rural extension services, and market access are necessary to support Turkish sheep producers.^24^^,^^25^ Native sheep breeds in Turkey have been selected over generations for local adaptation, demonstrating unique traits like disease resistance and climate resilience. However, indiscriminate crossbreeding and exotic breed replacement are eroding these native breeds’ genetic diversity. Molecular genetic analyses using techniques like microsatellites, SNPs and whole genome sequencing provide insights into the genetic makeup of these breeds. This information is crucial for conservation, genetic improvement, and managing genetic diversity.^26^^,^^27^ High-density SNP arrays offer a powerful means to analyse sheep breeds’ genetic diversity and population structure. These arrays, containing thousands to millions of SNPs, enable high-resolution genomic analyses in breeds worldwide. SNP genotyping helps understand population history, admixture, inbreeding and selection signatures. It is valuable for tracing historical origins, migrations, and identifying traits of economic interest.^28–30^ Most studies that have characterized Turkish sheep’s population structure and genetic diversity have utilized SSR markers.^25^^,^^31–34^ Additional research is needed to employ high-density SNP arrays for characterizing Turkish sheep’s genetic diversity. Such studies are pivotal in revealing more intricate details about the population structure and genomic variations, particularly those influencing local adaptation. A holistic approach incorporating geospatial, climatic, phenotypic and production system data could significantly enhance our understanding of these breeds. This comprehensive perspective is crucial for these sheep’s conservation, breeding strategies and sustainable management. This study aims to evaluate the population structure and genetic diversity of Turkish sheep and assess their kinship with sheep in nearby countries of Turkey.

## Materials and methods

This study involved the analysis of sheep genetic data, specifically employing the Illumina OvineSNP50 BeadChip as reported by Sempéré et al.^35^ Access to this data is provided through the WIDDE database, which is an online interface for next-generation exploration of genetic diversity. The dataset included a total of 219 animals from various breeds. The Turkish breeds were Karakas (KRS) with 18 animals, NDZ with 20 animals, and Sakiz (SKZ) with 22 animals. The Iranian breeds included Moghani (MOG) with 34 animals, Qezel (QEZ) with 35 animals and Afshari (AFS) with 37 animals. The Cyprus breed was the Cyprus Fat Tail (CFT) with 30 animals, and Greece breed Chios (CHI) with 23 animals. Genotypic quality control (QC) was applied to the merged genotypic data using PLINK.^36^ In QC, the samples and SNPs with missing genotype rates greater than 0.05 were discarded from the data set. The remaining missing SNPs were imputed with the Wright method using snpReady^37^ after excluding non-autosomal chromosomes in R Core Team.^38^ Afterward, the SNPs with minor allele frequencies (MAF) less than 0.01 and the SNPs not in Hardy–Weinberg equilibrium (HWE; *p* < 10^−6^) were removed from the genotype data set. After this filtering, a HWE test was applied to check whether the genotype frequencies of the loci fit HWE using the R package pegas.^39^ After QC, the imputed and cleaned genotypic data comprised 46,314 loci and 92,628 alleles for 26 chromosomes in 219 samples from eight sheep breeds.

### Statistical analysis

The R packages hierfstat^40^ and dartR^41^ were used to calculate the basic statistics consisting of individual counts, allelic frequencies, observed heterozygosities (Ho), expected heterozygosities (He), genetic diversities (Hs), allelic richness (AR) and the inbreeding coefficient (Fis) per locus and population. A re-randomization test with 1000 replications was applied to test the statistical significance of pairwise differences between the expected heterozygosities of the populations. An AMOVA was also applied using the poppr.amova function of the poppr package with the ade4 method. Population differentiation was further evaluated using pairwise Fst estimates according to the Euclidean method using the hierfstat package at the population level with 1000 permutations.

### Data visualization

SambaR R package was used to reveal the population structure and admixture of the examined sheep breeds. To determine the optimal number of genetic groups (clusters or K) for the eight sheep breeds, a cross-validation procedure was applied by changing Ks from 2 to 10.^42^ This was done by detecting the lowest value of the cross-validation error. According to the results, *K* = 3 gave the optimal number of ancestral. In this study, the bitwise.dist function of the poppr package^43^ was used for fast computation of the distances between the samples in a genlight object, then the poppr.msn function was run to construct MSNs using these distances. From the same package, the function plot_poppr_msn was used for plotting the phylogenetic trees. PCA was performed on the genlight object using the glPca function in the adegenet package^44^ in R. Additionally, the compoplot function of the adegenet package was utilized. The R package detectRUNS^45^ was executed to detect the Runs of Homozygosity (ROH) and inbreeding coefficient-based ROH (FROH) measures using the sliding-window option with the default parameter values provided in the package. In this package, the functions slidingRUNS.run and consecutiveRUNS.run were executed with the default values for the methods sliding-window-based run detection and consecutive SNP-based run detection, respectively.

## Results

### Analysis of genetic diversity

**Figure 2.**
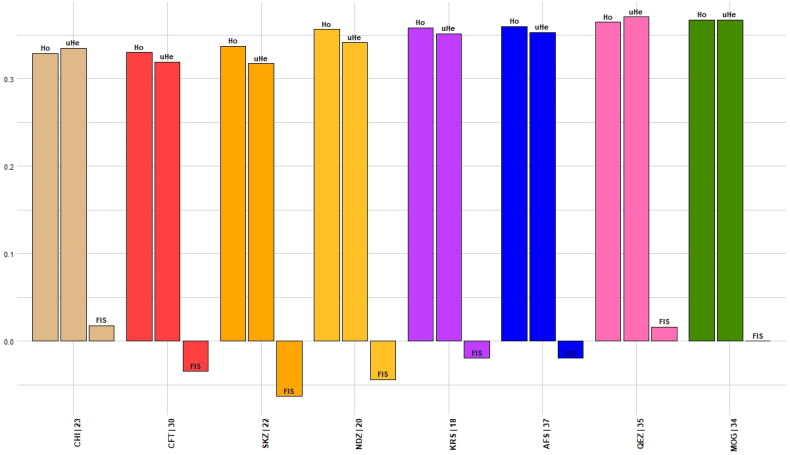
Barplots of Fis, Ho and uHe.

**Figure 3.**
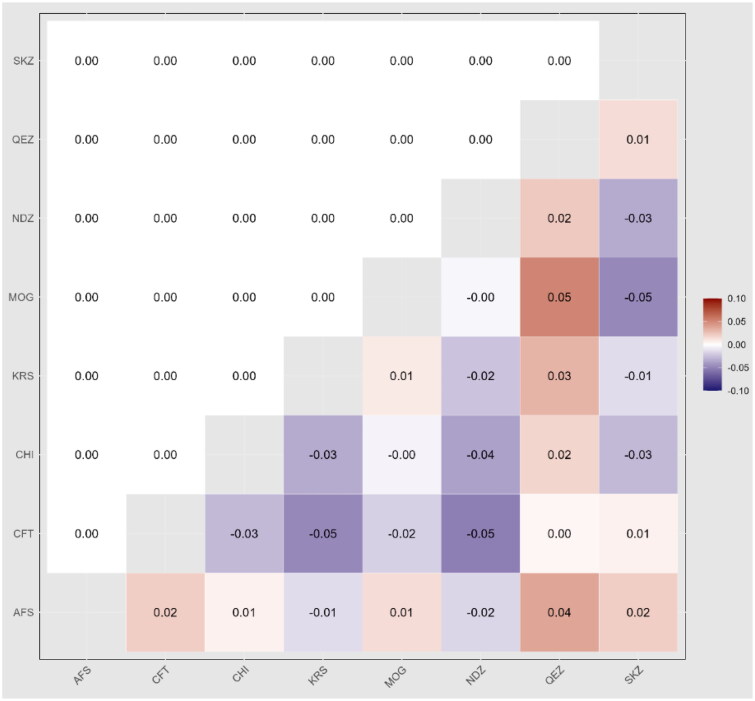
Heterozygosity among population.

The analysis of genetic diversity within and among the eight populations revealed moderate levels of diversity overall. The mean observed heterozygosity (Ho) ranged from 0.348 to 0.356, while the mean expected heterozygosity (He) ranged from 0.310 to 0.365, suggesting a deficit of heterozygotes in some populations. The AR ranged from 1.87 to 1.98, indicating moderately high numbers of alleles per locus. The total gene diversity (Ht) across populations was 0.374–0.379 and the corrected total gene diversity (Htp) was 0.377–0.384, indicating moderately high genetic variation in the overall sample. However, most of the diversity resided within populations rather than among populations, as evidenced by the low genetic differentiation (Fst = 0.079–0.080; corrected Fstp = 0.088–0.090). This was further supported by the low gene diversity among populations (Dst = 0.030–0.032; corrected Dstp = 0.035–0.036) compared to the total gene diversity. The Fis revealed varying levels of inbreeding among populations, with values ranging from −0.063 to 0.017 (Table 1; Fig. 2). Overall, these results demonstrate moderate genetic diversity both within and among these populations, with limited differentiation. Maintaining connectivity among populations will be important for conserving genetic variability in the future. The heterozygosity results show the heterozygosity values between the eight populations analysed (AFS, CFT, CHI, KRS, MOG, NDZ, QEZ and SKZ) (Fig. 3). Higher positive values indicate higher levels of heterozygosity or genetic diversity within a population. Overall, the populations show moderate to high heterozygosity, with values ranging from −0.052 to 0.052. The highest heterozygosity is seen in QEZ (heterozygosity = 0.052) and MOG (heterozygosity = 0.052), suggesting these populations have the highest genetic diversity. The lowest heterozygosity is observed in CFT (heterozygosity = −0.052) and NDZ (heterozygosity = −0.034), indicating lower diversity in these populations. While some variability in heterozygosity is seen between populations, the generally positive values suggest an overall high level of genetic diversity within the sample set. Further analysis of allele frequencies is needed to better understand the patterns of genetic variation between these populations.

**Table 1.** Genetic diversity analysis among the populations.

Population	Ho	He	uHe	Hs	Ht	Dst	Htp	Dstp	Fst	Fstp	Fis	Dest	AR
AFS	0.349	0.34	0.352	0.344	0.375	0.031	0.379	0.035	0.079	0.089	−0.019	0.057	1.96
CFT	0.351	0.313	0.319	0.345	0.376	0.031	0.38	0.035	0.079	0.088	−0.034	0.057	1.90
CHI	0.351	0.327	0.334	0.344	0.375	0.03	0.379	0.035	0.079	0.088	0.017	0.056	1.94
KRS	0.354	0.341	0.351	0.347	0.379	0.032	0.384	0.036	0.08	0.09	−0.019	0.059	1.95
MOG	0.349	0.361	0.367	0.343	0.374	0.031	0.379	0.036	0.08	0.09	−0.000	0.058	1.97
NDZ	0.356	0.332	0.341	0.343	0.374	0.031	0.378	0.035	0.079	0.089	−0.044	0.057	1.94
QEZ	0.351	0.365	0.370	0.345	0.376	0.031	0.38	0.035	0.08	0.089	0.016	0.057	1.98
SKZ	0.348	0.310	0.317	0.342	0.373	0.031	0.377	0.035	0.079	0.089	−0.063	0.057	1.87

Ho: observed heterozygosity; He: expected heterozygosity; Ht: overall gene diversity; Htp: corrected Ht; Dst: gene diversity among samples; Dstp: corrected Dst; Fst: fixation index; Fstp: corrected Fst; Fis: inbreeding coefficient; Dest: Jost’s D; AR: allelic richness

The Fis values and their corresponding confidence intervals reveal significant insights into these groups’ breeding patterns and genetic diversity. Each population exhibits distinct trends in inbreeding and heterozygosity, reflecting their unique genetic structures and breeding histories. For instance, the AFS population, with a Fis value of −0.02 and no variation in the confidence interval, displays a slight excess of heterozygosity, indicating an avoidance of inbreeding. This high precision in the estimate suggests a consistent genetic trend within the AFS population. Conversely, the CFT population, showing Fis values between −0.04 and −0.03, also indicates a trend toward heterozygosity but with a broader confidence interval, suggesting a stable yet slightly more variable genetic structure than AFS. The CHI population stands out with a consistent Fis value of 0.02, pointing toward a slight level of inbreeding. The uniformity in this interval indicates a precise estimate, emphasizing a slight but consistent inbreeding trend. Similarly, the KRS population mirrors the AFS trend, with a Fis value of −0.02 and no interval variation, indicating a stable genetic pattern with excess heterozygosity. The MOG population’s Fis value at 0.00 across the interval is particularly noteworthy, as it indicates genetic equilibrium and adherence to the Hardy-Weinberg principle, suggesting no significant inbreeding or outbreeding pressures. This contrasts with the NDZ population, where Fis values from −0.05 to −0.04 denote an apparent avoidance of inbreeding, with a narrow interval suggesting high precision in this trend. In the QEZ population, the Fis values from 0.01 to 0.02 indicate a slight inbreeding trend, a consistent pattern but not as pronounced as in other populations. Finally, the SKZ population shows a stronger tendency toward heterozygosity, with Fis values from −0.07 to −0.06, indicating a significant avoidance of inbreeding and a clear and consistent trend across this population.

### Genetic differentiation among the population

**Figure 4.**
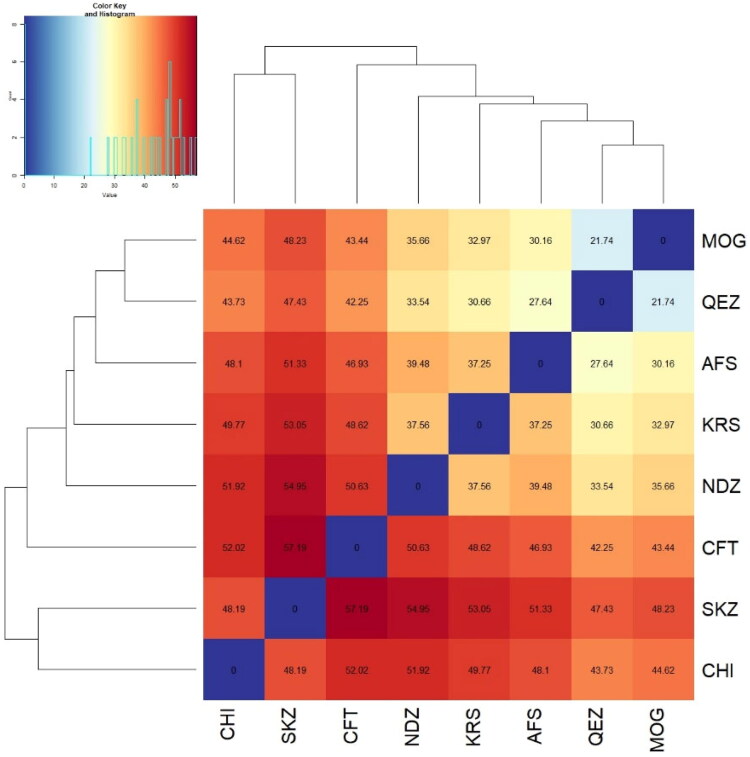
Heatmap of pairwise Euclidean values among the eight sheep breeds.

The Euclidean genetic distance values show the degree of genetic differentiation between the eight populations (AFS, CFT, CHI, KRS, MOG, NDZ, QEZ and SKZ). Larger distance values indicate greater genetic dissimilarity between populations. The overall distance values range from 21.74 to 57.19, suggesting moderate genetic differentiation among the groups analysed. The smallest distance is between MOG and QEZ (21.74), implying these two populations are most genetically similar. In contrast, the greatest distance is between CFT and SKZ (57.19), indicating these are the most divergent populations genetically. AFS and KRS show the next lowest distance (37.25), while CFT and NDZ have the second highest value (50.63). The remaining population pairs have intermediate distance values in the 40–50 range (Fig. 4). In summary, the genetic distance analysis reveals measurable but not extreme genetic differentiation between these eight populations. Closer analysis of the most genetically similar and distinct groups could provide insight into the evolutionary relationships among them.

### Analysis of Principal component

**Figure 5.**
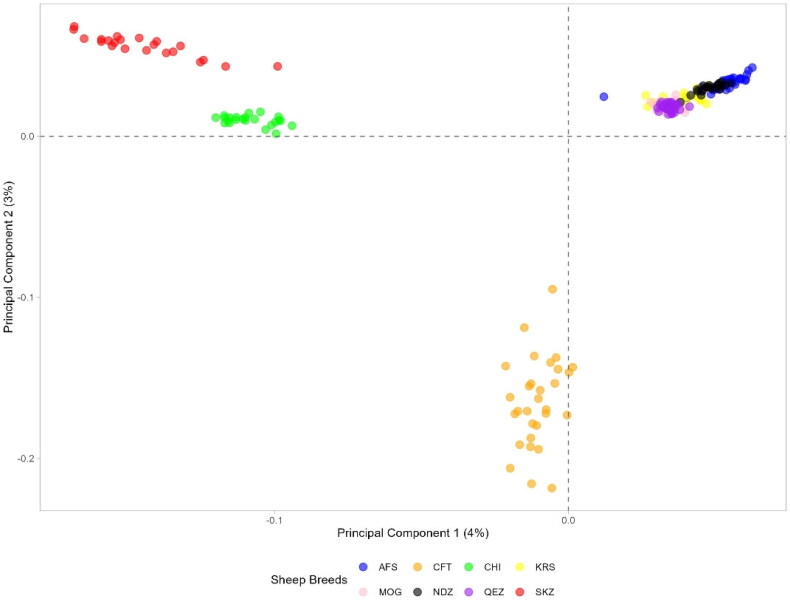
Principal component analysis for eight sheep breeds.

Principal component analysis (PCA) was carried out on genetic data from eight sheep breeds (AFS, CFT, CHI, KRS, MOG, NDZ, QEZ and SKZ) to assess genetic variation and relationships. The first two principal components (PC1 and PC2) accounted for 43.7% and 13.5% of the total variation, respectively. The PCA revealed distinct geographic positioning of the breeds that aligns with their genetic relationships (Fig. 5). The SKZ and CHI breeds clustered in close proximity, indicating their genetic similarity. The CFT breed occupied a solitary position away from the other breeds, consistent with its genetic uniqueness. The remaining breeds – NDZ, AFS, MOG, QEZ and KRS – formed an overlapping cluster, reflecting their closer genetic relationships. In particular, AFS, KRS, MOG, QEZ and NDZ clustered tightly together compared to the other breeds. This suggests strong genetic similarities exist. The geographic distribution of breeds in the PCA plot successfully captured the genetic relationships between the eight sheep populations analysed.

### Genetic structure analysis

**Figure 6.**
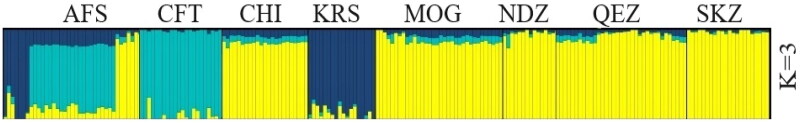
Admixture plot for eight breeds.

The admixture analysis was performed assuming 3 ancestral populations (*K* = 3). The analysis revealed three distinct genetic clusters representing ancestry components in the sheep breeds (Fig. 6). CFT Group, these sheep display a balanced genetic makeup between the three ancestral components. They inherit approximately 46% from the first component and 54% from the second component. This suggests a mixed heritage from both populations. CHI Group, most sheep in this group predominantly inherit from the first ancestral population, averaging 81%. The contribution from the second ancestral population is around 19%. This indicates a strong lineage from the first ancestral population with minor contributions from the second. KRS Group, these sheep have an overwhelmingly dominant contribution from the second ancestral population, averaging around 92%. The contribution from the first ancestral population is minor, averaging around 8%. SKZ Group, the sheep in this group majorly inherit from the first ancestral population, with an average contribution of around 96%. The second ancestral population’s contribution is minimal, averaging around 4%. QEZ Group, the sheep in the QEZ group predominantly inherit from the second ancestral population, with an average contribution of around 90%. The contribution from the first population averages around 10%. AFS Group, the sheep in this group have a dominant inheritance from the second ancestral population, averaging around 98%. The contribution from the first ancestral population is minimal, averaging around 2%. MOG Group, on average, sheep in this group inherit around 90% from the second ancestral population and around 10% from the first ancestral population. NDZ Group, most sheep in this group predominantly inherit from the second ancestral population, averaging around 98%. The contribution from the first ancestral population is minimal, averaging around 2%. While some breeds like CFT have a more balanced genetic makeup from both ancestral populations, others like SKZ and AFS have a dominant contribution from one specific ancestral population. These percentages provide a deeper understanding of each sheep breed’s genetic lineage and heritage.

### Analysis of molecular variance (AMOVA)

The AMOVA analysis partitioned the total genetic variation into among-population and within-population components. The among-population variation represents differences between populations, while the within-population variation represents differences between individuals within populations. The results show that differences among populations account for 3.16% of the total genetic variation, while differences within populations account for 96.84% (Table 2). The variance components indicate the amount of genetic variance at each level. The among-population variance component (0.0127) is lower than the within-population component (0.0276), reflecting the more significant proportion of within-population variation. The phi-statistic (PhiPT) of 0.316 indicates the degree of population differentiation. A higher PhiPT value indicates more significant differentiation between populations. Here, a moderate level of differentiation between populations was detected. The AMOVA results demonstrate that most genetic variation is found within populations rather than between populations in this sample. The populations show a moderate degree of genetic differentiation.

**Table 2.** Summary of AMOVA analysis.

Source	DF	SSD	MSD	Variance components (sigma2)	Phi-statistics	Variance coefficients
Among populations	7	2.6199	0.3742	0.0127	0.316	a: 27.119
Within populations	211	5.8242	0.0276	0.0276	–	–
Total	218	8.4441	0.4018	0.0403	–	–

MSD: mean squared deviation; SSD: sum of squares; DF: degree of freedom

### Phylogenetic analysis

**Figure 7.**
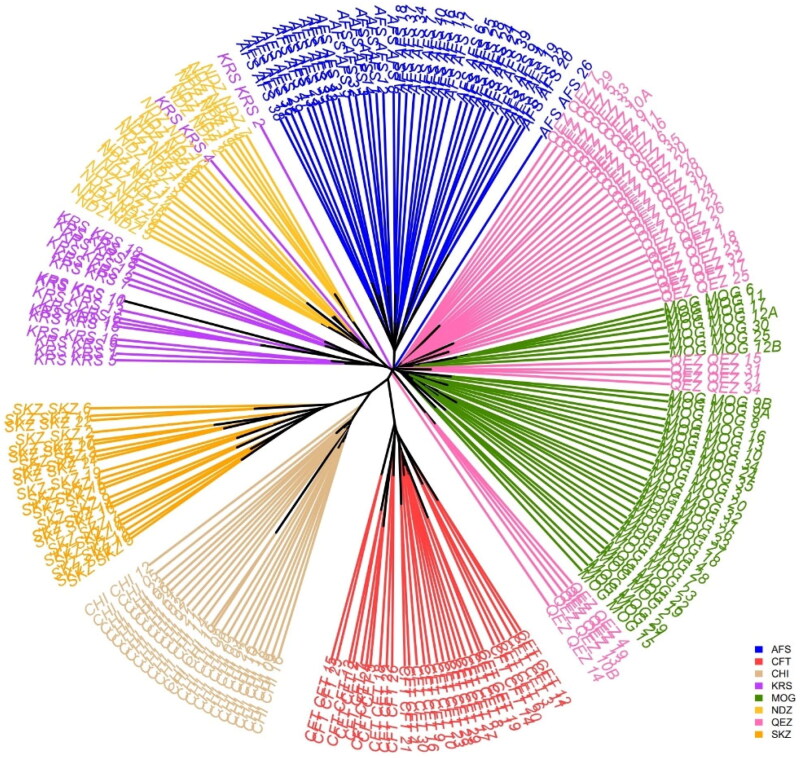
Comprehensive phylogenetic tree displaying genetic relationships.

The tree illustrates the genetic relationships and distances between eight sheep breeds based on Hamming distance (Fig. 7). The first distinct cluster consists solely of the SKZ and CHI breeds from Turkey and Greece. The early divergence of these two breeds indicates their substantial genetic differentiation from the other breeds analysed. As breeds from Turkey and Greece, respectively, their separation highlights geographic isolation and independent breeding histories that have shaped their genetic makeup. The second cluster contains only the CFT breed. The CFT groups separately from the Turkish and other breeds, likely reflective of its island origin and geographic isolation in Cyprus. The unique evolution of the CFT breed on the island setting has driven its genetic divergence from even the geographically proximate Turkish and Greek breeds. The third main cluster comprises the KRS, NDZ, AFS, MOG and QEZ breeds originating from Turkey, Iran and Greece. Within this cluster, further substructure is observed corresponding to geography. The Turkish breeds KRS, NDZ, MOG and QEZ form a sub-group, indicating their shared genetic ancestry and origins from within Turkey. The Iranian AFS breed also falls within this cluster but is the most divergent, reflecting its distinct Iranian origins and specialized traits. In summary, isolation on islands or within different countries has shaped the genetic profiles of these breeds. Those from nearby geographic regions show closer genetic relationships. The three main clusters demonstrate the influence of historical migrations, trading connections and separations in driving the genetic divergence between these sheep populations.

### Analysis of ROH and FROH

**Figure 8.**
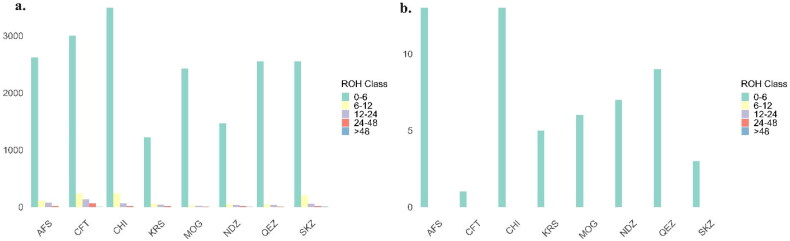
ROH distribution by breed, a) according to sliding homozygosity; b) according to sliding heterozygosity.

Sliding homozygosity and sliding heterozygosity were used to evaluate ROH and FROH. The sliding window method of homozygosity analysis revealed a variety of patterns across different size classes for the breeds studied. In the smallest ROH category (0–6 Mb), all breeds showed high levels of homozygosity, with MOG having the highest (0.97) and CFT the lowest (0.87) (Fig. 8a). When examining larger ROH categories (6–12 Mb and 12–24 Mb), a decrease in ROH percentages was observed across all breeds, suggesting a reduced incidence of longer homozygous segments. Notably, the Turkish breeds KRS, NDZ and SKZ showed unique patterns in these classes, with KRS and NDZ having similar percentages in the 6–12 Mb category, while SKZ had a slightly higher percentage. In the longest ROH category (>48 Mb), data was not available for AFS, CHI and KRS, while the other breeds had very low percentages, indicating rare occurrences of such extensive homozygous segments. The sliding window method of heterozygosity observed data for the 0–6 Mb ROH class showed a value of ‘1’ for each breed, which indicates complete homozygosity within this genomic range. This outcome suggests that within the 0–6 Mb genomic range, there is a complete absence of heterozygosity (or a 100% homozygosity rate) among the individuals sampled from each of these sheep breeds (Fig. 8b).

**Figure 9.**
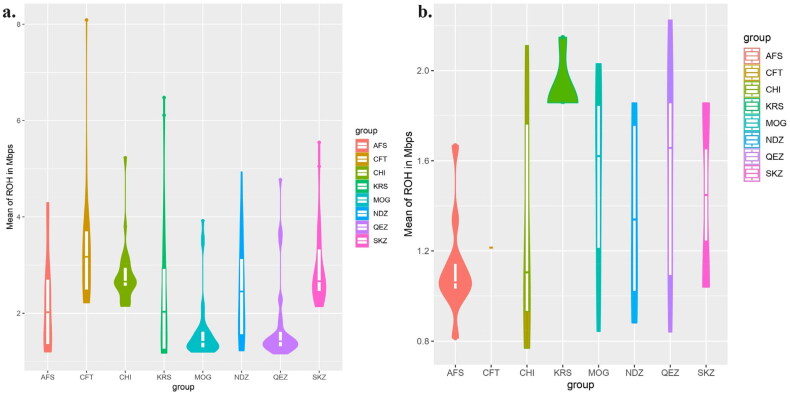
Comparative analysis of breeds based on mean ROH in mbps, a) according to sliding homozygosity; b) according to sliding heterozygosity.

The ROH analysis showed variability in homozygosity between the eight sheep breeds. The KRS, SKZ and NDZ breeds had the highest mean ROH percentages at 2.89%, 2.88% and 2.64%, respectively. The CFT breed had the next highest ROH at 3.48%, while the CHI and AFS breeds showed means of 2.79% and 2.40%. The QEZ and (MOG breeds displayed the lowest homozygosity with ROH of 1.81% and 1.79%. According to sliding heterozygosity, the results showed clear differences in ROH percentage between the eight sheep breeds analysed (Fig. 9a,b). The KRS had the highest mean ROH percentage at 2.00%. The MOG had the second highest mean at 1.46%, while the CHI and AFS breeds had means of 1.23% and 1.20%, respectively. The NDZ, SKZ, QEZ and CFT breeds had lower mean ROH percentages ranging from 1.20% to 1.42%.

**Figure 10.**
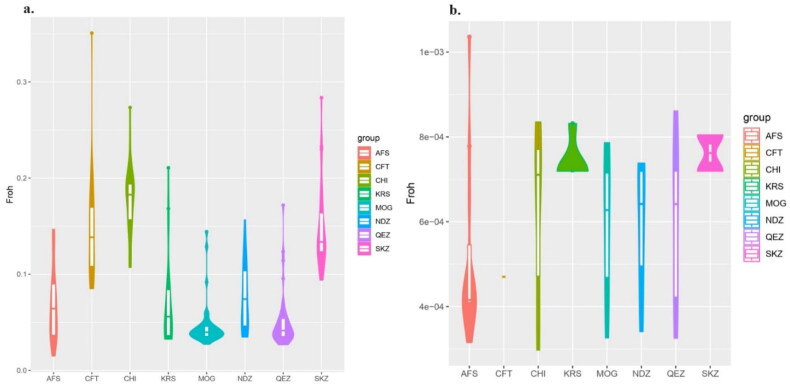
Comparative distribution of FROH means value among breeds, a) according to sliding homozygosity; b) according to sliding heterozygosity.

In the recent analysis of eight sheep breeds using the method of sliding homozygosity to calculate the FROH values, notable diversity was observed across breeds. The number of ROH segments varied, with the SKZ breed having the highest number of segments (22) and the KRS breed having the lowest (18). The minimum FROH values ranged from 0.01 in AFS to 0.10 in CHI. SKZ showed a high minimum value (0.09), indicating a greater level of homozygosity at the lower end of the spectrum compared to other breeds. The mean FROH values provide a measure of the average inbreeding within each breed; the highest mean value was found in SKZ (0.15), followed closely by CFT (0.14), with KRS (0.07) and NDZ (0.07) among the Turkish breeds showing lower mean values. The standard deviation (SD) of FROH values illustrates the variation within each breed, with CFT showing the largest variation (SD = 0.05) and MOG the smallest (SD = 0.02). The standard error of the mean (SEM) was relatively low for all breeds, suggesting a high precision of the estimated mean FROH values. The maximum FROH value was observed in CFT (0.35), indicating the presence of highly inbred individuals within this breed, while KRS showed the lowest maximum value (0.21) (Fig. 10a). The study has reported the FROH values of eight sheep breeds, focusing on the application of the sliding heterozygosity method. The number of ROH segments detected varied significantly among the breeds, with AFS having the most (11) and CFT the least (1). The minimum FROH values were zero for all breeds except for KRS and SKZ, both of which displayed a minimum value of 0.001. Interestingly, the mean FROH values were consistently low (0.001) across all breeds, with CFT having a mean of 0.000, which may indicate a very low level of inbreeding or an effective avoidance of inbreeding due to the breed’s management. There was no observed variation (SD = 0) in FROH within the breeds, which suggests a very narrow range of inbreeding coefficients among the individuals sampled. Similarly, the SEM was zero for all breeds, indicating uniformity in the FROH values across the samples. The maximum FROH value was 0.001 for all breeds, except for CFT, which had a maximum of 0.000 (Fig. 10b).

## Discussion

### Analysis of genetic diversity

The analysis of genetic diversity in KRS, SKZ and NDZ sheep provides valuable insights into the variation present within these Turkish breeds. KRS displayed relatively high heterozygosity (He = 0.357) and AR = 1.95, indicating substantial genetic diversity. This aligns with findings showing high diversity in KRS, likely reflecting its large population size and distribution across Turkey. SKZ exhibited slightly lower heterozygosity (He = 0.348) but high AR = 1.96, retaining considerable diversity despite its more regional breeding. NDZ showed heterozygosity (He = 0.355) on par with KRS yet lower AR = 1.87, suggesting higher diversity in the other Turkish breeds analysed. Nevertheless, NDZ harbors significant diversity overall. The notable diversity aligns with the sizeable historical population sizes and ancestral variation retained in these breeds.^4^ Proper monitoring and management of genetic variation will be crucial for sustaining the health and productivity of these Turkish genetic resources.^46^ The limited differentiation between breeds indicates relatedness and possible admixture.^47^ Controlled crossbreeding could assist in maintaining diversity while preserving unique ancestry.^11^ Overall, this analysis provides baseline insight into the population genetics of KRS, SKZ and NDZ sheep. Further genomic assessment on a broader sampling of Turkish breeds would enhance understanding of the relationships and distinctiveness of these regional breeds. The results can inform conservation efforts to safeguard the valuable diversity of these genetic assets.

### Genetic differentiation among the population

The KRS and AFS showed the lowest distance (37.25), indicating relative genetic similarity between these groups. In contrast, SKZ was most divergent from CFT based on the most significant distance (57.19). The Turkish breeds showed moderate differentiation, with distances ranging from 37.25 to 50.63 between population pairs. This aligns with the high within-breed diversity and evidence of admixture between breeds.^4^^,^^47^ The results establish a baseline understanding of population genetics for conserving these Turkish genetic resources. Proper monitoring and management of genetic variation will be critical to sustaining breed health and productivity.^46^ Further genomic assessment with a broader sampling would clarify the relationships and distinctiveness of these regional breeds. These findings can direct efforts to conserve the valuable diversity in KRS, SKZ and NDZ.

### Analysis of principal component

In the PCA, KRS clustered tightly with AFS, MOG, QEZ and NDZ, indicating genetic similarity between these groups. The proximity of the Turkish breeds KRS and NDZ aligns with evidence of admixture and shared ancestry among regional populations.^4^ SKZ occupied a more distinct position, clustering closely with CHI. This suggests that SKZ may harbor more unique diversity than KRS and NDZ. The PCA positions Turkish breeds across different plot areas, reflecting variability in their genetic backgrounds. Conservation efforts should consider maintaining this diversity within the breeds while sustaining breed health. Controlled crossbreeding programs could assist in balancing genetic uniqueness and vigor.^11^ The distinct position of SKZ also motivates further research into its origins and any unique adaptations. Overall, the PCA visualization provides valuable insights into the genetic relationships and distinctiveness of the Turkish breeds analysed. Incorporating genomic data from a broader sampling of breeds could further clarify these regional genetic resources’ relationships and ancestral makeup. These findings can help direct the management of genetic diversity within and among KRS, SKZ and NDZ sheep.

### Genetic structure analysis

The admixture analysis of Turkish sheep breeds KRS, SKZ, NDZ and others, assuming three ancestral populations (*K* = 3), provides a comprehensive view of these breeds’ genetic structure and diversity. This analysis is crucial in understanding these sheep breeds’ genetic composition and evolutionary history. The CFT group of sheep breeds exhibits a relatively balanced genetic makeup, inheriting approximately 46% from the first ancestral component and 54% from the second. This indicates a mixed heritage, which could result from historical migrations or breeding practices. This balanced genetic contribution aligns with findings from studies like those by Kalds et al. and Moradi et al.^48^^,^^49^ highlighting the complexity of sheep genetic evolution. The SKZ, AFS and NDZ groups show a high dominance of one ancestral population (ranging from 92% to 98% inheritance from a single population). This could indicate either a more isolated breeding history or strong selection pressures favoring genes from one population, as suggested in studies by Deniskova et al. and Mastrangelo et al.^50^^,^^51^ The significant differences in genetic makeup among these breeds underscore the importance of tailored conservation and breeding programs. As indicated by Djokic et al. and Gaspar et al.^47^^,^^52^ understanding the unique genetic makeup of each breed can aid in preserving genetic diversity and optimizing breeding strategies for specific traits. The Turkish breeds exhibit unique genetic profiles Compared to other sheep populations, as discussed in works by Getachew et al. and Kijas et al.^4^,^46^ This diversity is a testament to the region’s rich agricultural and breeding history and emphasizes the need for region-specific genetic studies. The distinct genetic clusters identified offer a valuable foundation for further research into the genetic basis of essential traits in sheep, as explored in studies by Davenport et al. and Zhou et al.^53^^,^^54^ This could lead to advancements in sheep breeding for traits like disease resistance, wool quality, and adaptability to climate change. The admixture analysis reveals significant genetic diversity and distinct lineage contributions among Turkish sheep breeds. This diversity reflects the complex history of sheep breeding in the region and serves as a vital resource for future breeding and conservation efforts. The findings underscore the importance of genetic studies in understanding breed-specific characteristics and making informed decisions for the sustainable management of genetic resources in sheep.

### Analysis ROH and FROH

The analysis of ROH and FROH in eight sheep breeds, with a particular focus on Turkish breeds KRS, SKZ and NDZ, provides critical insights into the genetic diversity and inbreeding status of these populations. This study’s findings offer valuable implications for conservation, breeding programs and understanding the genetic architecture of these breeds. High Homozygosity in Smaller ROH categories: The observation of high levels of homozygosity in the smallest ROH category (0–6 Mb) across all breeds indicates a general trend toward inbreeding or a historical bottleneck. This is consistent with the findings by Getachew et al.^46^ regarding Ethiopian sheep breeds. The decrease in ROH percentages in more significant categories (6–12 Mb and 12–24 Mb) across breeds suggests a reduced incidence of long homozygous segments. This could indicate relatively recent admixture events or effective breeding management practices aimed at minimizing inbreeding, as noted by Gaspar et al.^47^ The Turkish breeds (KRS, NDZ and SKZ) showed unique patterns, particularly in the 6–12 Mb ROH category. This could reflect breed-specific historical breeding strategies or genetic drift events, echoing similar observations in Mediterranean sheep breeds by Chessari et al.^28^ The significant variability in FROH values across the breeds, with breeds like SKZ showing higher mean values, indicates varying levels of inbreeding. The high FROH in SKZ might suggest either a closed breeding system or a small enough population size, as discussed in studies on genetic diversity by Deniskova et al.^50^ The complete homozygosity observed in the 0–6 Mb genomic range for all breeds suggests an intense selection pressure or founder effect in these regions, as also noted in genetic studies by Djokic et al.^52^ The consistently low mean FROH values across all breeds, particularly in CFT, suggest effective management strategies to avoid inbreeding or inherent genetic characteristics of these breeds that resist high inbreeding levels.

## Conclusions

This study provides important insights into the genetic diversity, structure and relationships among sheep breeds from Turkey, Iran, Cyprus and Greece. The moderate levels of genetic diversity observed indicate that these populations still harbor valuable genetic variation that should be conserved. At the same time, evidence of inbreeding in some breeds underscores the need for genetic management to avoid further erosion of diversity. The genetic distances and clustering patterns highlight the connectedness and differentiation among these sheep breeds, likely reflecting the combined influences of ancestry, selection and geographic isolation. While most genetic variation resides within populations, the inter-population differences are sufficient to reveal regional genetic relationships and structures. The ancestral component and phylogenetic analyses further elucidate the genetic lineages inherited by these populations from presumed ancestral gene pools. The distinctive ROH profiles among breeds showcase unique genomic patterns of homozygosity that provide signatures of selection and drift. Overall, these findings can inform breeding and conservation programs aimed at preserving the genetic uniqueness and integrity of these sheep breeds while also sustaining opportunities for future improvement and adaptation. The genetic resources of breeds like SKZ, NDZ and KRS should be monitored and managed to maintain diversity, health and product quality. This study thus provides a valuable genetic resource that enhances our understanding of sheep biodiversity in the Eastern Mediterranean region. Future research could build on these fundamental insights into genetic structure to identify functional variation related to phenotypic traits and adaptive potential in these breeds.
